# Correspondence

**Published:** 1880-07

**Authors:** 


### CORRESPONDENCE.

Office of Geo. L. Parmele, M. D., 25 Pratt St.,
Hartford, Conn., June 14, 1880.

*B. M. Wilkerson, M. D.,*
*Jr. Editor "Independent Practitioner."*

Dear Sir:—Thinking some of your readers may be interested in the following ancient bit of advice, I send it for you to use as you think best. Very truly yours, Geo. L. Parmele.

Extract from "*The Birth of Mankinde*, otherwise named, *The Woman's Booke.* Set foorth in English by Thomas Raynalde. Phisition and by him corrected, and augmented—Whose contenents yee may reade, in the table folowyng. but most playnely in the prologue—

Imprinted at London by Richarde Watkins.
Cum Privilegio 1568–
(Black letter)"

"To keepe and preserve the teeth cleane.

*First* if they be very yellow and filthie, or blackish, let a Barber scoure, rub, and pick them cleane, it shalbe very good to rub them every day with the root of a mallow, and to pick them cleane that no meate remayne and putrifie between the teeth.

*Item*, take of the small white pibble stones which be found by the water sydes, and beate them in very small powder, hereof take an ounze, and of masticke one dram, mingle them togeather, and with this powder once in X days rub exactly your teeth, and this shall keepe your teeth fayre and white: but beware yee touch not ne vexe the gummes therewithall.

*Item.* to stable and steadfast the teeth, and to keep the gummes in good case, it shall be very good every day in the morning to wash well the mouth with red wine."

---

San Francisco, Cal., June, 1880.

Dear Editors:

I send you the following for The Independent Practitioner. S.

Post Obstetrical Colloquy.

Mother.—But Doctor, have not the instruments injured my child's jaw?

Doctor.—Not a bit of it, Madam! *I* was delivered with forceps. Indeed, *all our great men* have been of late years!

Mother.—But, will it come back into shape again?

Doctor.—Oh, the vis naturæ gynocologæ will restore it.

Mother.—But my other children were born without forceps, and it has been scarcely an hour since my first pain. Those who attended me with them did not think artificial aid necessary.

Doctor.—O, my dear Madam, there has been a *great advance* since then in labor-saving manipulations, for *we* have now a better *appreciation of time.* You certainly cannot fail to appreciate the manly appearance of your child's head from the operation.

